# G-quadruplex structural motifs modulate protein–RNA interactions within the transcriptome

**DOI:** 10.1186/s13059-025-03795-0

**Published:** 2025-09-30

**Authors:** Uditi Bhatt, Cameron W. Evans, Anne Cucchiarini, Julien Gros, K. Swaminathan Iyer, Jean-Louis Mergny, Nicole M. Smith

**Affiliations:** 1 School of Molecular Sciences, The University of Western Australia, Crawley, WA 6009 Australia; 2 ARC Training Centre for Next-Gen Technologies in Biomedical Analysis, The University of Western Australia, Crawley, WA 6009 Australia; 3 School of Pharmacy and Pharmacology, University of Tasmania, Sandy Bay, TAS 7005 Australia; 4 Laboratoire d’Optique et Biosciences, École Polytechnique, CNRS, INSERM, Institut Polytechnique de Paris, 91120 Palaiseau, France

**Keywords:** G-quadruplex, RNA secondary structure, Fused in sarcoma, FUS, G4 RIP-seq

## Abstract

**Background:**

RNA secondary structures, including G-quadruplexes (G4s), have emerged as vital players in protein–RNA interactions. The RNA-binding protein Fused in Sarcoma (FUS), which is strongly implicated in both neurodegenerative disease and cancer, is known to interact with RNA molecules through a variety of GU-rich sequences. However, a definitive consensus motif for FUS–RNA recognition and binding has not yet been determined. Here, we hypothesize that G4 structures, which are inherently G-rich, may play a key role in FUS binding.

**Results:**

We examine the role of G4s in FUS–RNA binding by developing an RNA immunoprecipitation sequencing (RIP-seq) protocol under G4-stabilizing and non-stabilizing conditions. We find that G4s regulate the binding of FUS to target RNAs, providing new information on protein–RNA binding motifs, while reinforcing the importance of RNA secondary structures as pivotal regulators of protein interactions.

**Conclusions:**

These insights advance our understanding of FUS–RNA binding dynamics and future potential for identifying new therapeutic targets for neurodegenerative disease and other FUS-related pathologies.

**Supplementary Information:**

The online version contains supplementary material available at 10.1186/s13059-025-03795-0.

## Background

RNA-binding proteins (RBPs) are the primary force modulating the life cycle of RNAs, ranging from transcription, splicing, post-transcriptional modifications, and degradation. These protein–RNA interactions are determined by recognition features within the RNAs, including sequence motifs, secondary structures, and biochemical modifications. Importantly, RNA secondary structures are increasingly identified as key players in RBP–RNA interactions; thus, investigating the roles of different RNA structures in protein binding is essential to understanding their cellular functions.

Fused in sarcoma (FUS) is a ubiquitous and highly expressed RNA/DNA-binding protein involved in a variety of cellular processes including RNA processing, cell proliferation, genome integrity, and transcriptional regulation. Importantly, mutations in FUS have been linked to the development of neurodegenerative diseases, particularly amyotrophic lateral sclerosis (ALS), as well as certain cancers including sarcoma, pancreatic cancer, and lung cancer [1–8]. Disease-associated mutations in FUS are known to alter the subcellular distribution and RNA binding of the protein, as well as promote liquid–liquid phase separation (LLPS) leading to the formation of abnormal FUS aggregates and contributing to disease pathogenesis and progression [9–11]. Identifying the driving factors that govern FUS–RNA interactions will help to characterize the molecular mechanisms and critical binding events by which FUS exerts its cellular functions.

Composed of 526 amino acids over 15 exons, FUS represents a promiscuous protein with extensive RNA binding interactions in the cellular context. FUS consists of a low-complexity QGSY-rich region at the N-terminus, which is involved in LLPS and interactions with other proteins [12, 13]. At the C-terminus, RGG-rich domains, an RNA recognition motif and a zinc-finger motif make up the nucleic acid binding module of the protein, of which the RNA recognition motif binds to RNA with broad specificity and the zinc-finger specifically binds to GGU motifs [14]. Multiple groups have investigated the sequence motifs involved in FUS–RNA target recognition for over two decades with results from different methodologies including SELEX, NMR, and CLIP-seq converging on GUGGU, GGUG, GGU, or GU-containing sequences; however, no consensus sequence motif has been discerned to date [15–20].

In addition to sequence-based recognition, recent studies have identified FUS specificity towards RNA structure, representing novel binding mechanisms [14, 21, 22]. For example, FUS has been shown to bind to non-canonical secondary structures known as G-quadruplexes (G4s) [23]. G4s form in certain G-rich regions of DNA and RNA when guanine bases self-assemble through Hoogsteen hydrogen bonding into multiple planar tetrads known as G-quartets. The three-dimensional G4 structure consists of stacked G-quartets, stabilized by central cations, generally monovalent, which confer different levels of stability to the structure (K^+^ > Na^+^ > Li^+^) depending on the ionic radius relative to the size of the negatively charged core created by the O6 atoms [24, 25]. FUS interactions with RNA G4 (rG4) structural motifs have been increasingly investigated in recent years; however, the interactions identified remain limited to a few specific examples [23, 26–28]. In particular, the RGG domain of many proteins, including FUS, is known to bind to rG4s with high affinity, including G4s found in *Telomeric Repeat-Containing RNA (TERRA)* and neuronal mRNAs, with functional implications for the regulation of telomere length, histone methylation, and neural function [23, 26, 29–32]. Furthermore, several other well-characterized FUS binding partners such as *NEAT1* and *MALAT1* are known to contain rG4 structures; however, whether FUS binds to the G4 structure in these RNAs rather than G-rich sequences, or through another binding mechanism, is unknown [33, 34]. Overall, the impact of rG4 structures on endogenous FUS binding to target RNAs remains poorly explored, especially at the transcriptome-wide scale.

In this study, we developed a G4-focused version of RNA immunoprecipitation sequencing (G4 RIP-seq) using total RNA from SH-SY5Y human neuroblastoma cells and recombinant C-terminal FUS protein under G4-stabilizing and non-G4-stabilizing conditions. We identify rG4s as important elements that modulate FUS binding to RNA targets throughout the transcriptome, furthering understanding of FUS–RNA target recognition and representing unique structural features that may be targeted to control FUS function in disease states such as ALS.

## Results

**Fig. 1:**
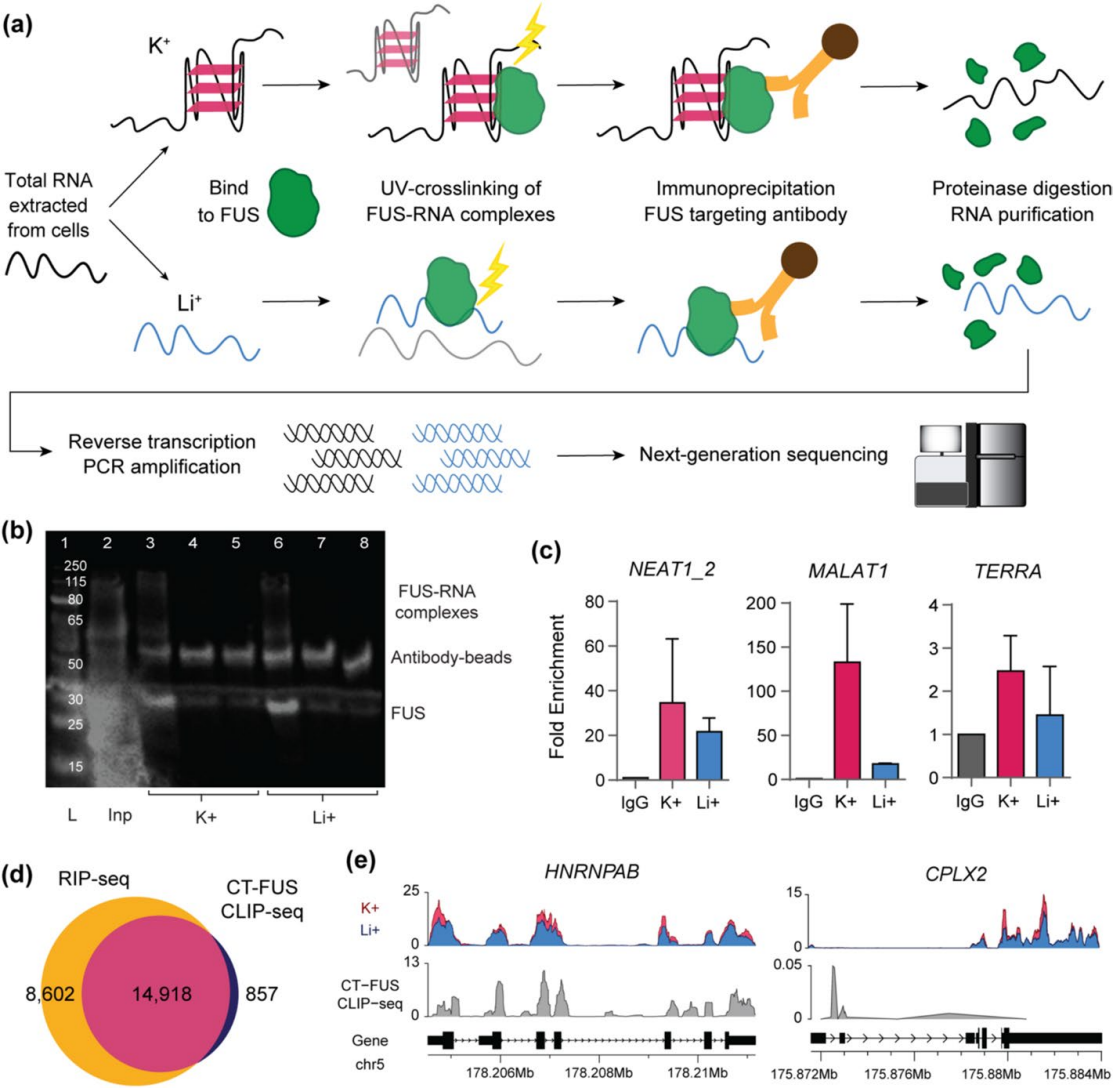
G4 RIP-seq protocol and overlap with CLIP-seq data.** a** Schematic of the G4 RIP-seq protocol. G-quadruplex shown in pink, FUS protein in green, antibody in orange, and magnetic bead in brown. **b** Western blot showing successful immunoprecipitation of FUS-RNA complexes in K^+^ and Li^+^ buffers. Lanes: 1, Ladder (L); 2, Input (Inp); 3–5, FUS-RNA IP in K^+^ with His_6_ antibody, IgG, or no antibody, respectively; 6–8, FUS-RNA IP in Li^+^ with His_6_ antibody, IgG, or no antibody, respectively. **c** RT-qPCR of *NEAT1_2*, *MALAT1*, and *TERRA* from isolated RNA following IP of FUS-RNA complexes in K^+^ and Li^+^ conditions with IgG or His_6_-specific antibody. Note the differences in Y-axis values between the three sequences. **d** Overlap of genes between G4 RIP-seq and CLIP-seq data, with > 10 counts per million across all samples. **e** Karyoplots showing BigWig coverage of representative genes in G4 RIP-seq with overlap (*HNRNPAB*, left) and without overlap (*CPLX2*, right) with C-terminal FUS CLIP-seq (grey). Genomic region shown in black. G4 RIP-seq in K^+^ shown in pink and Li^+^ in blue

To identify G4-associated FUS interactions with RNA, we performed RIP-seq under G4-stabilizing (K^+^) and non-G4-stabilizing (Li^+^) conditions (Fig. 1a). Total RNA was extracted from SH-SY5Y neuroblastoma cells and secondary structures were annealed in either G4-stabilizing (K^+^) or non-G4-stabilizing (Li^+^) buffer. RNA was then bound and UV-crosslinked to a His_6_-tagged recombinant FUS protein (Additional file 1: Fig. S1) containing the RNA-binding C-terminal domains (aa 269–526). The LLPS propensity of FUS is known to influence its ability to bind RNA. Given that RNA is known to buffer LLPS with different effects on binding depending on the concentration, it is difficult to separate these two properties of FUS [35, 36]. To minimize the effects of this in our experiments, all experiments were carried out using the C-terminal domains of the FUS protein, excluding the low-complexity domain that has previously been reported to be largely responsible for LLPS in FUS [36], and the amount and concentrations of RNA, as well as temperature conditions, for all samples in our G4 RIP-seq experiments were normalized.

The His_6_-tagged FUS-RNA complexes in K^+^ or Li^+^ buffer were immunoprecipitated (IP) using an antibody specific to the His_6_ tag, which was confirmed by Western blot with comparisons against IgG-IP and no antibody negative controls (Fig. 1b). Immunoprecipitated RNAs were purified and converted to cDNA for RT-qPCR analysis, showing enrichment of known RNA targets of FUS in IP over the IgG-IP negative control (Fig. 1c). cDNA libraries of purified RNAs were then prepared for next-generation sequencing to determine the effects of G4 structures throughout the FUS-bound transcriptome. cDNA libraries of Input samples, which underwent all experimental steps of the RIP process except for immunoprecipitation of the FUS-RNA complexes, were simultaneously prepared and sequenced.

In total, 23,520 unique genes were detected from the G4 RIP-seq samples with > 10 counts across the K^+^ and Li^+^ IP samples, with high reproducibility between replicates (Additional file 1: Fig. S2). 25,928 genes were identified in the Input samples, indicating that RNAs from ~ 90.7% of genes found in SH-SY5Y cells bind to FUS. The G4 RIP-seq genes were also compared to CLIP-seq data of a C-terminal (aa 242–526) FUS construct in the same SH-SY5Y cell line [33]. In total, 14,918 (95%) genes from the CLIP-seq dataset were detected in our G4 RIP-seq experiment (Fig. 1d), indicating a strong correlation between our results and the FUS–RNA interactions measured within a cellular environment. An additional 8602 genes were detected in the G4 RIP-seq that were not present in the CLIP-seq data, indicative of potential RNA binding partners that may be spatially or temporally separated from FUS within SH-SY5Y cells under the given CLIP-seq experimental conditions. Representative examples of genes in which the G4 RIP-seq data overlaps or does not overlap with genes with CLIP-seq peaks are shown in Fig. 1e.

**Fig. 2:**
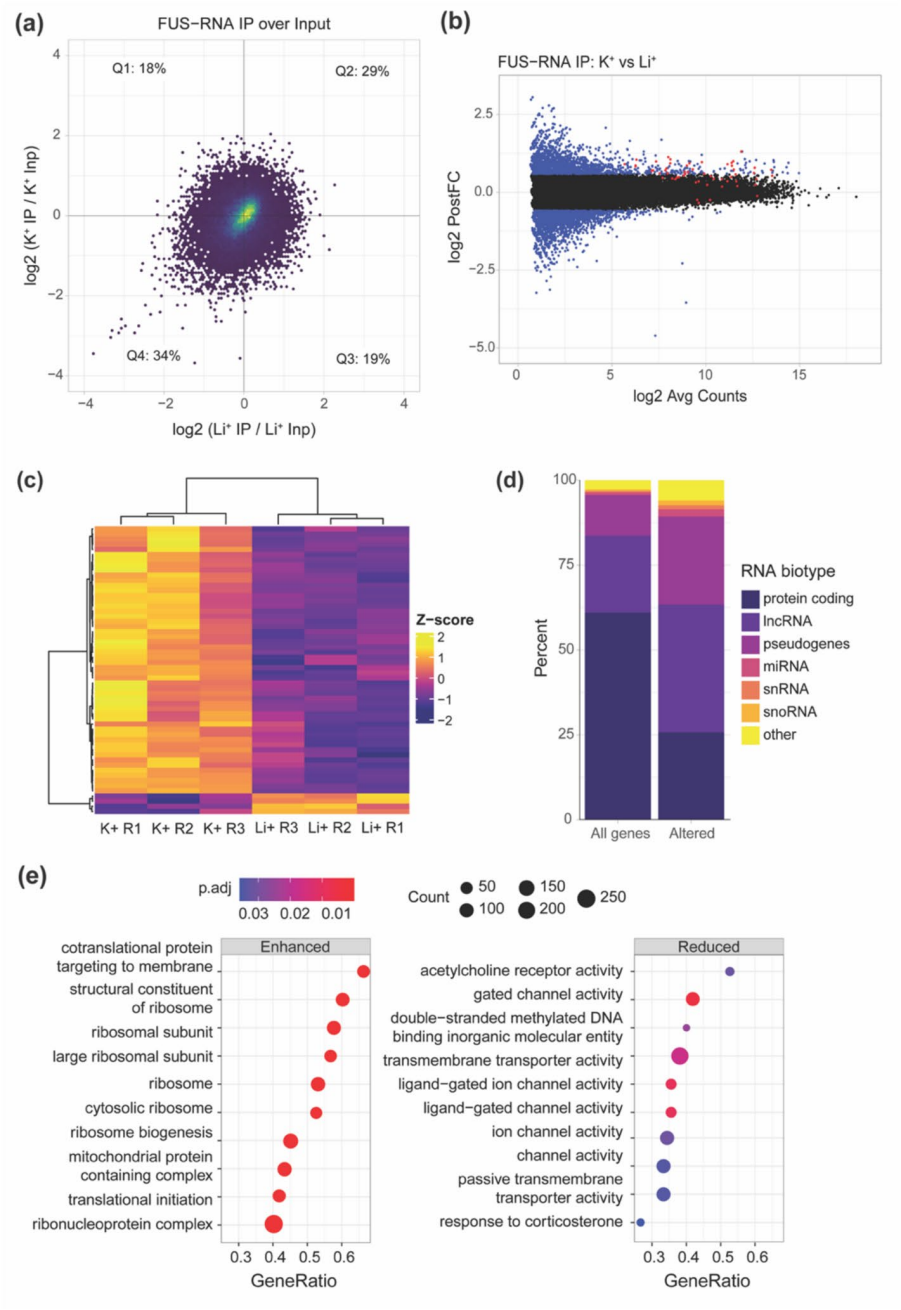
G4 RIP-seq shows differential immunoprecipitation of FUS-RNA targets between K^+^ and Li^+^ conditions. **a** Quadrants plot showing log_2_PostFC between K^+^ IP and K^+^ Input over log_2_PostFC between Li^+^ IP and Li^+^ Input. **b** Mean difference (MD) plot showing log_2_PostFC between K^+^ and Li^+^ IP samples over log_2_ average counts. Red points indicate significantly different genes (FDR < 0.05), whereas blue points represent genes with a minimum absolute log_2_PostFC of 0.585, corresponding to a 1.5-fold increase or decrease, but without statistical significance (FDR > 0.05). Black points represent unaltered genes. **c** Heatmap of significantly differential genes between K^+^ and Li^+^ IP. R1, R2, and R3 = biological replicate 1, 2, and 3, respectively. Heatmap of all genes with a minimum absolute log_2_PostFC of 0.585 between K^+^ and Li^+^ IP is provided in Additional file 1: Fig. S5. **d** Bar graph showing difference in RNA subtype populations between all FUS-bound genes detected in the IP dataset (All genes) vs “altered” genes. **e** Dot plot showing top 10 GSEA pathways in which FUS binding is significantly (*padj* ≤ 0.05) enhanced (left) or reduced (right) by K^+^ in comparison to Li^+^

To examine the global effects of rG4 formation on FUS binding, K^+^ and Li^+^ IP samples were first compared to their corresponding Inputs (Fig. 2a). 17.6% of genes were enriched in the K^+^ but not Li^+^ IP samples indicating RNAs in which G4 formation enhances FUS binding. For 19.5% of genes, IP was weaker in K^+^ than Li^+^, possibly representing RNAs for which G4 formation appears to strongly hinder interaction with FUS. 29.2% of genes were enriched in both K^+^ and Li^+^ IP samples signifying strong FUS binding, and 33.7% of genes had lower normalized counts in IP than the Input showing poor FUS binding to these RNAs. No major differences were observed between the K^+^ and Li^+^ input samples (Additional file 1: Fig. S3). Next, we investigated the differential immunoprecipitation of genes specifically between the K^+^ and Li^+^ conditions to obtain a more detailed view of G4-associated IP. 3,337 genes had a minimum 1.5-fold change (|log_2_PostFC|> 0.585), hence termed “altered” RNAs (Fig. 2b), and 56 genes were statistically significant (FDR < 0.05). Of these top 56 differential genes, 52 had increased IP under the K^+^ condition (Fig. 2c), suggesting that K^+^, which favors G4 formation, may enhance FUS binding to target RNAs in comparison to the absence of G4s, as in the Li^+^ condition, through a structurally dependent mechanism. Previously, an inverse relationship between FUS binding affinity and RNA length has been identified, and thus short sequences may show unexpectedly high enrichment regardless of structure. For genes with FDR < 0.05 and |log_2_PostFC|> 0.585, we observed no significant correlation between the RNA gene length and enriched immunoprecipitation (log_2_PostFC) between the K^+^ and Li^+^ conditions (Additional File 1: Fig. S4), indicating that our differential binding patterns reflect G4-dependent structural effects rather than length-dependent binding preferences.

Next, we wanted to explore if G4s affected FUS binding to RNAs with specific functions in cells. Initial examination of RNA subtypes revealed a disproportionate number of non-coding RNAs (ncRNA) represented in the “altered” set of RNAs in comparison to the complete set of FUS-bound, immunoprecipitated RNAs, including long non-coding RNAs (lncRNAs), pseudogenes, and microRNAs (miRNAs) (Fig. 2d). This was followed by gene set enrichment analysis (GSEA) of the G4 RIP-seq data, which showed a significant increase in FUS binding to RNAs belonging to pathways associated with ribosome synthesis and translation, and a decrease in binding to RNAs associated with the activity of ligand-gated ion channels, particularly the acetylcholine receptor (Fig. 2e). Pathway associations were more statistically significant for the RNAs that showed increased binding to FUS in the presence of G4 structures (Fig. 2e, left) than for RNAs that had reduced FUS binding (Fig. 2e, right).

To further examine the relationship between the RIP-seq results and G4 structures, we predicted the formation of G4s throughout the transcriptome using the G4-iM Grinder algorithm and compared the number of predicted G4s (pG4s, with a threshold of G4-iM Grinder Score ≥ 20 keeping only stable G4s with medium to high G4-forming potential and excluding sequences with low G4-forming potential) in each RNA relative to its differential FUS binding between K^+^ and Li^+^ conditions. pG4s on the complementary strands were not considered. A W-shaped distribution was observed for the mean number of pG4s *vs* fold change, with peaks seen at the center and both ends of the fold change axis (Additional File 1: Fig. S6a). The G4-iM Grinder algorithm additionally provides a score parameter, indicating the likelihood of a G4 structure forming from the sequence motif identified; however, only slight variation in pG4 score was seen over fold change (Additional file 1: Fig. S6a). No difference was seen between the number of genes with at least one pG4 between the significant genes set and all genes, nor in the pG4 content within the groups for the K^+^ or Li^+^ conditions (Additional file 1: Fig. S6b). Importantly, the number of pG4s identified can only provide an indication and does not directly correspond to the in vitro or in vivo formation of G4s, nor inform the functional relevance of the structures.

**Fig. 3:**
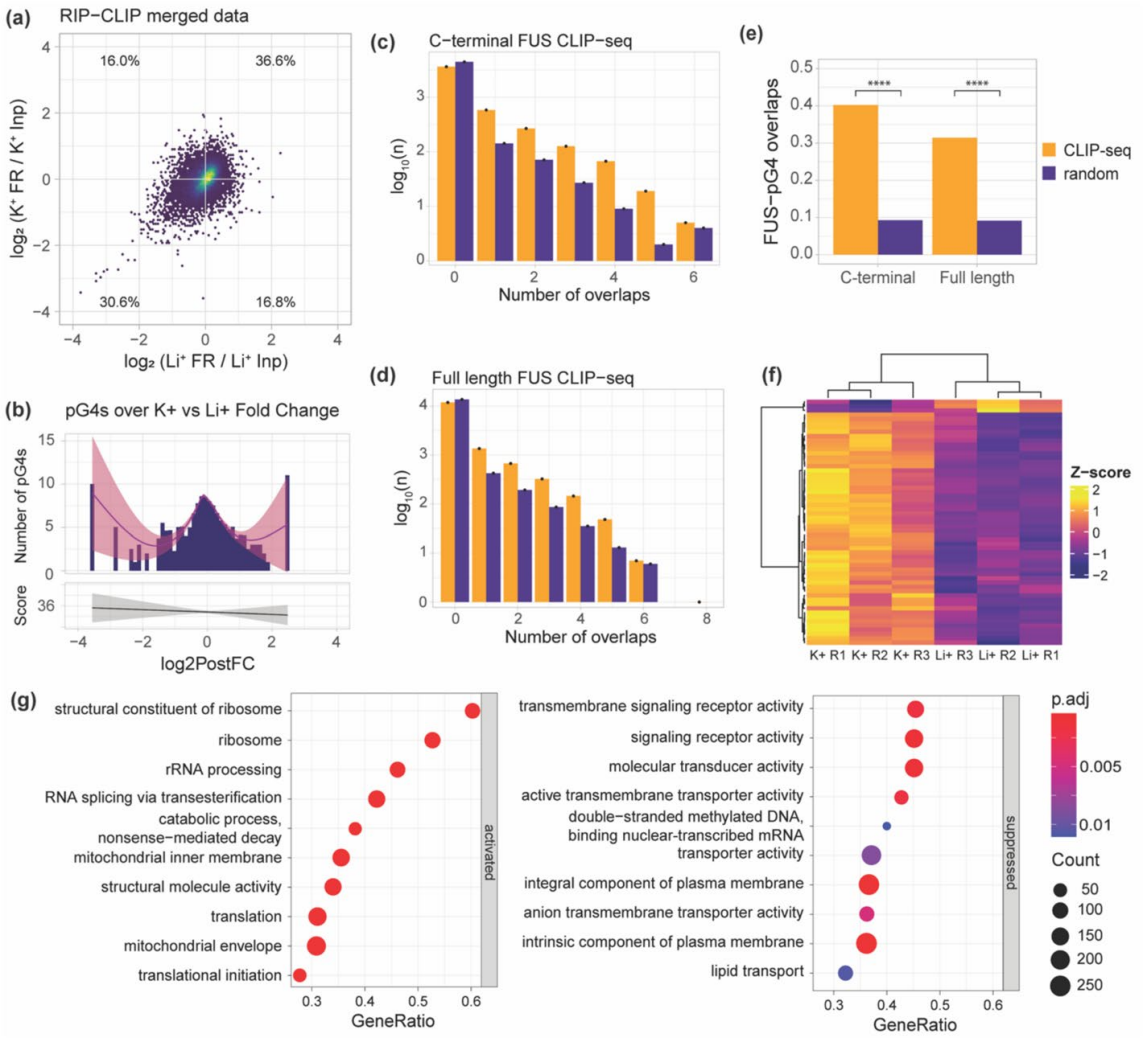
G4 RIP-seq genes and pG4s in CLIP-seq data.** a** Quadrants plot for genes in both G4 RIP-seq and CLIP-seq datasets showing log_2_PostFC between K^+^ IP and K^+^ Input over log_2_PostFC between Li^+^ IP and Li^+^ Input. **b** Mean number (above) and score (below) of pG4s over log_2_PostFC between K^+^ and Li^+^ IP for genes in both RIP-seq and CLIP-seq datasets. **c** Log_10_ number of genes over number of pG4 overlaps with C-terminal FUS binding sites from CLIP-seq (orange) compared to overlaps when binding sites are randomly distributed across the genome (purple). **d** Log_10_ number of genes over number of pG4 overlaps with full-length FUS binding sites from CLIP-seq (orange) compared to overlaps when binding sites are randomly distributed across the genome (purple). **e** Mean number of pG4 overlaps with all CLIP-seq binding sites for C-terminal and full-length FUS (orange) compared to overlaps when binding sites are randomly distributed (purple). Comparison of means *t*-test, ****p ≤ 0.0001. **f** Heatmap of significantly differential genes between K^+^ and Li^+^ IP samples that are present in both G4 RIP-seq and C-terminal FUS CLIP-seq datasets. R1, R2, and R3 = biological replicate 1, 2, and 3, respectively. **g** Dot plot showing top 10 GSEA pathways significantly (*padj* ≤ 0.05) activated or suppressed by K^+^ in comparison to Li^+^ in genes present in both G4 RIP-seq and C-terminal FUS CLIP-seq datasets

We then considered only the FUS-bound genes that were also found to be FUS bound in SH-SY5Y cells from CLIP-seq results [33]. A noticeably more elongated shape is seen in the quadrants plot (Fig. 3a). Similar to the full range of FUS-bound RNAs, the number of pG4s over K^+^ vs Li^+^ fold change had a W-shaped distribution; however, it had a much more pronounced center peak and reduced peak at K^+^ enriched, higher fold changes that correspond to G4-enhanced FUS binding (Fig. 3b).

To determine if G4s act as direct targets for FUS or affect FUS binding by altering the overall structure of the RNA, we examined overlaps with pG4s from the G4-iM Grinder transcriptome-wide predictions. Positive overlap results were defined as pG4 motifs intersecting within a 100 bp window either side of the FUS binding sites, following the definition of a FUS-bound region of RNA in the CLIP-seq dataset [33]. In comparison to random shuffling of FUS binding sites across the transcriptome, both C-terminal and full-length FUS binding sites were found to be significantly enriched at pG4 regions (Fig. 3c–e). The number of RNAs with significantly altered IP, proportion of RNAs for which FUS binding is enhanced under K^+^ conditions, and enriched pathways from GSEA were similar to the G4 RIP-seq only dataset (Fig. 3f, g).

**Fig. 4:**
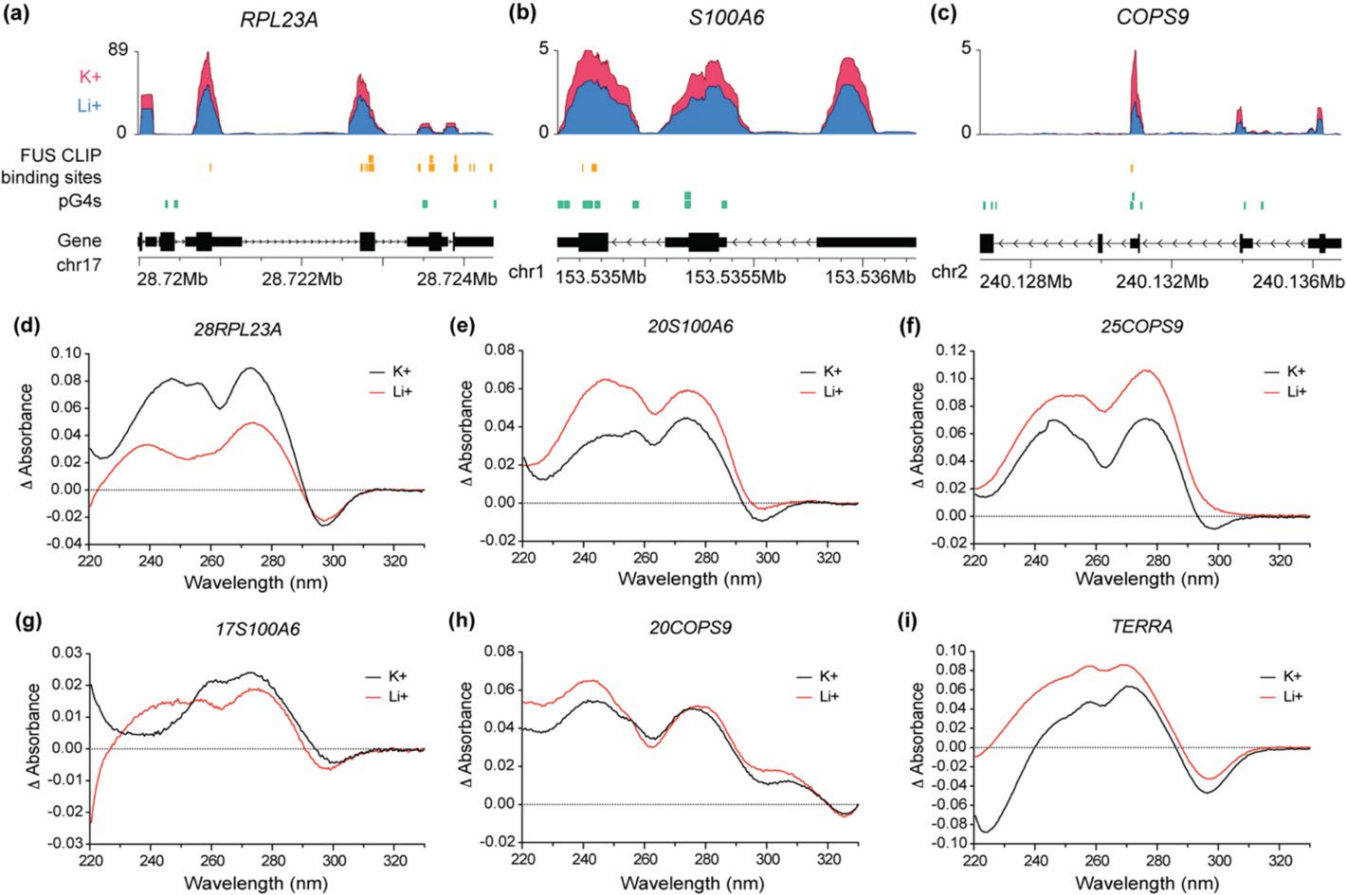
pG4s in K^+^ enriched RNAs align with FUS binding sites and form G4s in vitro. Karyoplots showing BigWig coverage of K^+^ (pink) and Li^+^ (blue) G4 RIP-seq results, FUS binding sites from CLIP-seq (yellow), and pG4 motifs (green) over the entire gene (black) for **a** *RPL23A*, **b** *S100A6*, and **c** *COPS9*. Thermal difference spectra (TDS) of **d** *28RPL23A*, **e** *20S100A6*, **f** *25COPS9*, **g** *17S100A6*, **h** *20COPS9* RNA pG4s, and **i** *TERRA* positive control G4, in K^+^ (black) and Li^+^ (red) buffer conditions.

To validate the formation of G4 structures, five pG4 sequences were selected from three unique RNAs based on the significant enrichment of the RNA in the K^+^ IP condition and overlap with FUS binding sites identified in the CLIP-seq data (Fig. 4a–c). Specifically, pG4s within *RPL23A*, *COPS9*, and *S100A6* (Additional file 1: Table S1) were examined using circular dichroism (CD), thermal difference spectra (TDS), and recognition by G4-specific fluorescent molecules Thioflavin-T (ThT) and *N*-methyl mesoporphyrin IX (NMM). These RNAs play critical roles in essential cellular processes, namely ribosome synthesis, protein ubiquitination, and cell proliferation and differentiation, respectively [37–40]. All experiments were performed under the same K^+^ and Li^+^ buffer conditions as used for the G4 RIP-seq alongside non-G4 forming RNAs *SNORA23* and *PDHX* as negative controls, and *TERRA* as a positive control (Additional file 1: Table S1) [26].

**Fig. 5:**
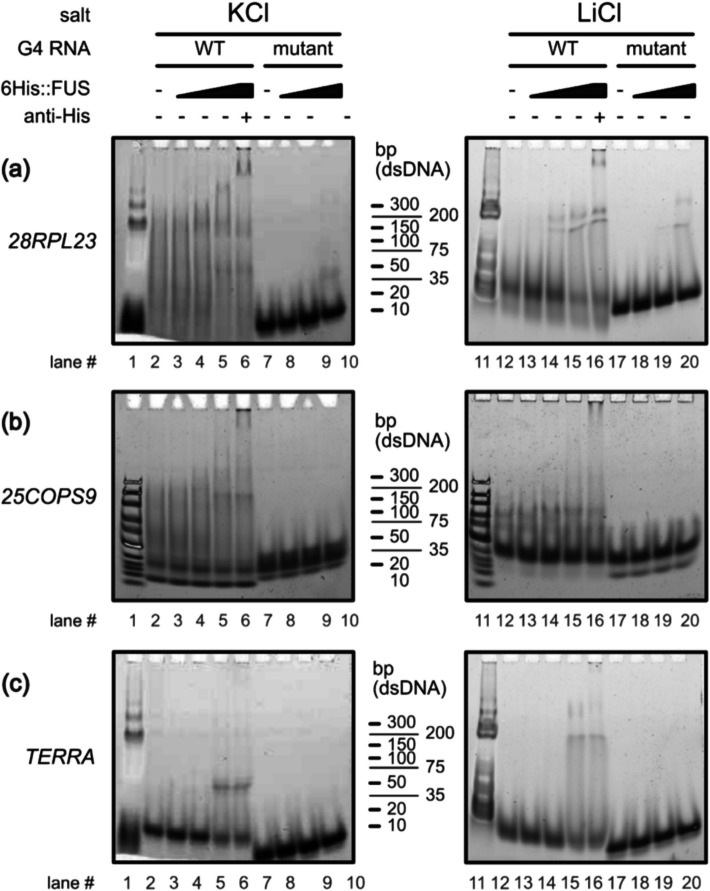
RNA binding to FUS is dependent on G4 formation. EMSAs of **a** *28RPL23*, **b** *25COPS9*, or **c** *TERRA* positive control WT or mutant RNA annealed in either KCl (left) or LiCl (right) containing buffer, incubated with increasing concentrations of recombinant His-tagged FUS protein. Anti-His_6_ antibody was added to WT samples in both KCl and LiCl buffers as the supershift condition

TDS analysis confirmed G4 formation in K^+^ conditions for *28RPL23A* (Fig. 4d), *20S100A6* (Fig. 4e), and *25COPS9* (Fig. 4f) sequences, with characteristic peaks at ~ 245 nm and 275 nm and a trough at ~ 295 nm, whereas the TDS spectra for *17S100A6* (Fig. 4 g) and *20COPS9* (Fig. 5 h) pG4 sequences were relatively poor, lacking the 245 nm peak and 295 nm trough, respectively [41]. TDS of the well-documented lncRNA G4, *TERRA*, was used as a positive control (Fig. 4i). Under Li^+^ conditions, the 245 nm peak is shifted back for *28RPL23A*, and the 295 nm trough is lost or reduced for *25COPS9* and *20S100A6*, respectively, all indicating poor G4 formation. The *17S100A6* sequence in Li^+^ displayed a characteristic G4 spectrum, indicating the rG4 can form even in the absence of a stabilizing cation such as K^+^, illustrating that the most stable G4 structures only need minute amounts of stabilizing cations. This was not observed for the *20COPS9* sequence, with the Li^+^ condition lacking the 295 nm trough similar to the K^+^ condition. TDS of mutants in which the central guanine residues were converted to uracil, preventing G4 formation, were confirmed to not display the characteristic G4 spectra (Additional file 1: Fig. S7) [41].

All selected pG4 oligos displayed parallel G4 topology in K^+^ buffer as observed by CD, with a peak at ~ 260 nm and a trough at ~ 240 nm, whereas the spectral profile in Li^+^ showed reduced signal intensity [42]. CD spectra of negative controls *SNORA23* and *PDHX* displayed peaks at ~ 220 and ~ 270 nm indicative of single-stranded RNA (Additional file 1: Fig. S8). Additionally, the signal intensity of G4-specific fluorescent molecules ThT and NMM in fluorescence assays was notably higher for WT pG4 RNAs in comparison to non-G4 forming mutants (Additional file 1: Fig. S9). A comparison between K^+^ and Li^+^ was not performed in this case as both ThT and NMM are known to induce and stabilize G4 structures.

To validate G4 structure-dependent RNA binding to FUS, *28RPL23A* and *25COPS9* RNAs were further examined with electrophoretic mobility shift assays (EMSAs) with increasing concentrations of His_6_-tagged recombinant FUS protein, alongside non-G4-forming mutants, in KCl- and LiCl-containing buffers (Fig. 5). FUS-RNA complex formation, indicated by decreased mobility through the gel, was observed in both KCl and LiCl conditions for WT, but not mutant forms, of *28RPL23A* (Fig. 5a), *25COPS9* (Fig. 5b), and the positive control *TERRA* (Fig. 5c). These FUS-RNA complexes were also observed with greater intensity compared to unbound RNA in the KCl condition than LiCl for *28RPL23A*, *25COPS9*, and *TERRA*, indicating stronger affinity for FUS-RNA binding in the presence of a stable G4 structure. In addition, a supershift assay condition was included by adding an anti-His antibody to the WT RNAs tested at the highest FUS concentration, with significantly reduced mobility seen (Fig. 5a–c, lanes 6 and 16).

## Discussion

In this study, we explored the roles of G-quadruplex non-canonical secondary structures as a mechanism for FUS protein recognition and binding to its target RNAs in SH-SY5Y neuroblastoma cells. We developed a G4-focused RIP-seq protocol using G4-stabilizing (K^+^) and non-G4-stabilizing (Li^+^) buffer conditions and examined differential FUS-RNA binding. RNAs from over 3000 genes were differentially precipitated between K^+^ and Li^+^ conditions, indicating large-scale rG4-mediated FUS binding. In addition, RNAs from ~ 20,000 genes were precipitated in both K^+^ and Li^+^, highlighting the multiple mechanisms beyond rG4s by which FUS can bind to RNA as reported in the literature. In total, 1061 RNAs were enriched in the K^+^ condition after immunoprecipitation, representing a subset of RNAs for which G4 structures may play a role in FUS recognition and binding. In comparison, 1064 RNAs were enriched in the Li^+^ condition, representing RNAs for which G4 formation in the K^+^ condition may hinder protein binding, and thus FUS-linked immunoprecipitation of the RNA is only seen in the non-G4-stabilizing Li^+^ buffer condition. Of the 56 significantly differential RNAs immunoprecipitated between K^+^ and Li^+^ conditions, 52 (93%) were enriched in K^+^ and 45 (80%) contained a pG4 motif (with G4-iM Grinder score ≥ 20). The RNAs that did not contain a pG4 were primarily pseudogenes and may be forming intermolecular rG4s with other RNA molecules that assist FUS binding or are co-immunoprecipitated with other FUS–RNA binding partners. While the majority of G4s are unlikely to form in Li^+^, highly stable rG4s may appear in Li^+^ conditions with reduced stability and can require micromolar amounts of favorable cations to fold as a result of residual K^+^ from the RNA purification or FUS protein purification steps. This is also reflected in the biophysical studies and EMSA experiments, where FUS-RNA binding is observed in both the K^+^ and Li^+^ conditions to different extents. Our EMSA validation confirms that G4s act as direct binding sites. For RNAs enriched in K^+^ conditions, G4 stabilization enhances FUS binding. For RNAs enriched in Li^+^ conditions, the reduced G4 stabilization may make alternative FUS binding sites more accessible that would otherwise be sequestered or structurally occluded when G4s can form. Consequently, we see a combination of both G4-dependent and G4-independent binding mechanisms in our G4 RIP-seq results.

A 95% overlap was observed between our results and RNAs immunoprecipitated in C-terminal and full-length FUS CLIP-seq data available in the same SH-SY5Y cell line, indicating excellent representation of FUS-RNA interactions in cells with G4 RIP-seq [33]. Importantly, the CLIP-seq provided critical data regarding specific positions of FUS binding sites and was used to examine enrichment of binding sites at pG4-containing regions of the transcriptome. We observed strong overlap between pG4s and FUS binding sites which was significantly enriched over random distribution of FUS binding sites throughout the transcriptome, indicating that G4s are likely structural targets for FUS. Given that individual RNA molecules can contain multiple FUS binding sites, the G4 structures may only be involved in FUS binding to a subset of these sites. Stabilizing or destabilizing the G4 may result in FUS preferentially binding to alternate, structured regions on the RNA molecule and is particularly relevant given the dynamic nature of G4s within cells. This concept coincides with previous studies demonstrating complex structural rearrangement of FUS domains and higher-order multimerization of the protein upon RNA binding [22, 43]. Additionally, FUS binding can remodel RNA structure, including resolving rG4s, to enable other RNA-binding domains of the protein to interact with the molecule [14, 22, 44]. While we have focused on G4 structures, FUS recognition and binding to target RNAs is multifactorial and context-dependent, as multiple types of secondary structures, in addition to sequence, can contribute to this process. Here, we experimentally demonstrate that for G4-dependent RNAs, G4 structures, and not the G-rich sequence, are direct binding targets of FUS, as binding is lost when the G4 is unable to form, supporting previous results of G4-dependent FUS binding to other RNAs [32, 44].

GSEA of the G4 RIP-seq data showed a significant increase in FUS binding to RNAs associated with ribosome synthesis and translation, including *RPL23A*. Previously, FUS has been shown to regulate translation, including by association with polyribosomes through mammalian target of rapamycin (mTOR) kinase signaling activity, repeat-associated non-AUG (RAN) translation through the G4 structures of the C9orf72 repeat sequence in familial ALS, and local translation at axons of motor neurons [3, 45–47]. Furthermore, a decrease in mRNAs encoding ribosomal proteins was observed in transgenic ALS mice harboring FUS mutations [48]. Our results indicate previously unknown roles of rG4s in FUS interactions with ribosomes as a novel mechanism of FUS-associated translational regulation.

Similarly, we identified *S100A6* and *COPS9* as RNAs with significant enrichment in the G4-stabilizing K^+^ immunoprecipitation samples. The S100A6 protein was previously identified to be one of the most significantly downregulated proteins upon FUS knockdown in motor neuron-like murine NSC-34 cells and human HEK-293 T cells, resulting in impaired cell proliferation [49]. *COPS9* encodes subunit 9, also known as CSNAP, of the highly evolutionarily conserved COP9 signalosome complex, which plays important roles in cell homeostasis by regulating protein ubiquitination [38, 50]. Some evidence of FUS protein association with subunits 5 and 6 of the COP9 complex has been shown through affinity capture mass spectrometry; however, functional implications of this and FUS interactions with other subunits are not well characterized [51]. These RNAs, namely *RPL23A*, *S100A6*, and *COPS9*, selected from those with the most significantly different FUS binding upon G4 formation, were confirmed to form G4 structures using biophysical assays, providing an additional level of verification to our study. Formation of the G4 structure was confirmed to be critical for the FUS-RNA binding interaction as determined by EMSA, further supporting the results of our G4 RIP-seq experiment.

Conversely, RNAs that preferentially bound to FUS in the absence of a G4 structure were found to be associated with ligand-gated ion channels and the acetylcholine receptor. Previous studies have shown FUS binding to and regulating the transcription of key ligand-gated ion channels including acetylcholine, glutamate, and γ-aminobutyric acid (GABA) neurotransmitter receptors in the brain [34, 52]. Additionally, ALS mouse models with mutant FUS commonly display altered levels of RNAs encoding ion channels and solute carrier (SLC) transporters essential to neurotransmitter signaling [48]. This study identifies G4 structures as a regulatory mechanism for FUS binding to RNAs essential for cell–cell signaling and synaptic function.

## Conclusions

In summary, we for the first time have identified the full range of G4-dependent FUS–RNA interactions throughout the transcriptome by developing a G4-specific RIP-seq protocol that takes advantage of the differential formation and stability of G4 structures in K^+^ and Li^+^ cations. G4 RIP-seq data displayed high agreement with FUS CLIP-seq performed in SH-SY5Y cells examining interactions that can occur within a cellular environment, with the advantage of identifying all possible FUS–RNA targets. In addition, specific positions of FUS binding sites from the CLIP-seq data closely correlated with pG4s, indicating that rG4s are a likely structural binding motif. This study provides essential information in understanding the role of RNA secondary structure and FUS–RNA interactions in biology, particularly for neurodegenerative diseases, and identifies rG4s as unique targets for the development of novel therapeutics for FUSopathies.

## Methods

### Cell culture

SH-SY5Y cells (obtained from and authenticated by ATCC, CRL-2266) were cultured in DMEM/F-12 (1:1) media (Gibco, 11320-033) supplemented with 10% fetal bovine serum (FBS, Gibco, 10099-141) under standard culture conditions (37 °C, 5% CO_2_) and passaged at ~ 80% confluency. Cells used for all experiments were between passage 3 and 9. Cells were confirmed to be mycoplasma-free.

### Recombinant FUS protein expression

His_6_-tagged FUS protein construct pET28a-TEV-FUS containing the RNA-binding module (aa 269–526) was transformed into competent Rosetta2 (DE3) *E. coli* and expressed in LB media + 50 µg/mL kanamycin + 0.1 mM ZnCl_2_. One liter of cultures were grown at 37 °C for 3 to 4 h (OD_600_ 0.6–0.8) before inducing protein expression overnight at 22 °C with 0.1 mM isopropyl β-D-1-thiogalactopyranoside (IPTG). Cells were collected by centrifugation (5000* g*, 10 min, 4 °C) and pellets were stored at − 80 °C.

Pellets were resuspended and cells lysed in 40 mL ice-cold lysis buffer (50 mM Tris pH 8 + 1 M NaCl + 2 mM β-mercaptoethanol (β-ME) + 0.5% v/v Triton X-100 + 5 mM imidazole + 50 µM ZnCl_2_) to which 10 mg lysozyme and 1 × EDTA-free Protease Inhibitor Cocktail (Roche, 04693132001) were added. Lysates were treated with DNase I, sonicated, and clarified by centrifugation (24,000* g*, 45 min, 4 °C). Ni–NTA beads pre-equilibrated in lysis buffer were added to clarified lysates and protein was bound for 30 min at 4 °C with gentle rotation. Beads were transferred to a column and washed with 50 mM Tris pH 8.0 + 1 M NaCl + 2 mM β-ME + 10 mM imidazole before eluting FUS protein in 50 mM sodium phosphate pH 8.0 + 1 M NaCl + 2 mM β-ME + 200 mM imidazole + 10 µM ZnCl_2_ at a slow drip rate. The eluate was dialyzed in pre-soaked 10 kDa MWCO dialysis tubing overnight at 25 °C against 50 mM Tris pH 8.0 + 2 mM β-ME + 10 µM ZnCl_2_. Protein production and purification was confirmed by SDS-PAGE (Additional file 1: Fig. S1) and concentration measured using UV absorbance at A_280_ (Nanodrop), and aliquots stored at -80 °C until required.

### G4 RIP-seq

Total RNA from SH-SY5Y cells grown to ~ 80% confluency in a 10 cm dish was extracted using the RNeasy Mini Kit (Qiagen, 74106) with RNase-free DNase set (Qiagen, 79254). Purified RNA was standardized to 4 µg per sample in 200 µL K^+^ or Li^+^ RIP buffer (150 mM KCl or LiCl, 25 mM Tris pH 7.4, 5 mM EDTA, and 100 U/mL SUPERase-In added fresh before use), denatured for 5 min at 95 °C, and G4 structures annealed by slowly cooling to 4 °C at 1 °C/min. Protein A Dynabeads (Thermo Fisher, 10002D) were blocked with 0.1 mg/mL bovine serum albumin (BSA) and 0.1 mg/mL cold water fish skin gelatin (Sigma, G7041) in phosphate-buffered saline + 0.1% v/v Tween-20 (PBST) overnight at 4 °C with gentle rotation.

2 µg per sample of His_6_-tag antibody (Thermo Fisher, MA1-21,315) in PBST was conjugated onto the blocked beads for 1 h at 4 °C, gentle rotation; 2 µg mouse IgG (Thermo Fisher, 10400 C) in PBST was used as an isotype control. PBST only (no antibody) and RNA only (no FUS) negative controls were also included. 1 × Complete Protease Inhibitors Cocktail (Roche, 04693132001) was added to all samples. Annealed RNA was incubated with recombinant FUS protein for 2 h at 4 °C with gentle rotation at a 1:1 (w/w) ratio before crosslinking on ice with 254 nm UV light at 500 mJ/cm^2^. Crosslinked FUS-RNA complexes were immunoprecipitated at 4 °C overnight with gentle rotation. Unbound protein and RNA were removed with 3 × 5 min washes in ice-cold K^+^/Li^+^ RIP wash buffer (RIP buffer with 0.5 mM DTT, 0.5% NP-40 (Thermo Fisher, 85124), and 1 × Complete Protease Inhibitors Cocktail (Roche, 04693132001) added fresh before use. Five percent of the resuspended beads were taken at the second wash step for SDS-PAGE analysis. Samples were reverse crosslinked with 1 mg/mL proteinase K (New England Biolabs, P8107S) in proteinase K reaction buffer (100 mM Tris, pH 7.5, 50 mM NaCl, 1 mM EDTA, 0.2% SDS) for 2 h at 55 °C, 1200 rpm. Released RNA was purified with neutral phenol–chloroform (Thermo Fisher, AM9732) in 2 mL Heavy Phase Lock Gel (HPLG) tubes (QuantaBio, 2302830) and precipitated overnight at − 20 °C in 4 volumes ethanol, 0.1 volume 5 M NaCl, and 3 µL linear acrylamide (Thermo Fisher, AM9520). Precipitated RNA was pelleted by centrifugation at 16,000* g* for 45 min at 4 °C, washed twice with 75% ethanol, air-dried, resuspended in 15 µL RNase-free water, and quantified with Qubit RNA HS assay kit (Thermo Fisher, Q32852). Input samples were subject to all the same steps as FUS-RNA IP samples except for immunoprecipitation and subsequent washing steps. Successful pull-down was confirmed with Western blot and RT-qPCR.

cDNA libraries of FUS-RNA IP and Input samples from three independent biological replicates were prepared using the NEBNext Ultra II Directional Library Prep Kit for Illumina (New England Biolabs, E7765) with the NEBNext rRNA Depletion Kit (New England Biolabs, E7405) and Unique Dual Index Primer Pair Multiplex Oligos (New England Biolabs, Index Primers Set 1, E6440). cDNA libraries were sequenced on a HiSeq Xten 150 bp PE (Illumina) by Macrogen Oceania.

### Western blot

FUS-RNA complexes before and after immunoprecipitation were resolved on a 12% SDS-PAGE alongside normal mouse IgG isotype control and no antibody controls at 120 V. Protein-RNA complexes were transferred to nitrocellulose membranes and blocked with TBST (20 mM Tris pH 7.6 + 150 mM NaCl + 0.1% v/v Tween-20) + 5% w/v skim milk powder for 30 min at 25 °C. Blocked membranes were incubated with rabbit anti-FUS antibody (Sigma Aldrich, PLA0083) diluted to 0.1 µg/ml (1/10,000) in blocking buffer overnight at 4 °C, then washed 3 × 5 min with TBST. Goat anti-rabbit HRP-conjugated secondary antibody (Abcam, ab205718) in blocking buffer was added to a final concentration of 0.2 µg/ml (1/10,000 dilution) and membranes were incubated for 2 h at 25 °C, protected from light. Membranes were washed 3 × 5 min with TBST before incubating with ECL reagents (Merck Millipore, P90720), protected from light. Bands were visualized using chemiluminescence (Biorad ChemiDoc).

### RT-qPCR

RNA levels of known G4-forming lncRNA binding partners of FUS, *NEAT1_2*, *MALAT1*, and *TERRA* were measured in K^+^ and Li^+^ conditions with RT-qPCR against mock-IP IgG controls using the QuantiTect Reverse Transcription Kit (Qiagen, 205313) and QuantiNova SYBR Green RT-PCR Kit (Qiagen, 208156) following manufacturer’s instructions on a LightCycler 480 Real-Time PCR machine (Roche). Sequences of primers used are provided in Additional File 1: Table S2.

### G4 RIP-seq data analysis

Raw G4 RIP-seq reads were mapped to the human reference genome GrCh38 and Ensembl gene annotations version 90 using STAR version 2.7.6a. BAM files were converted to BigWig files using bedtools. Abundance estimates were computed with HTSeq version 2.0.2. All downstream analyses were performed in R. Genes with low expression (< 10 counts across all samples) were removed and the filtered set of genes was used for differential gene expression analysis using EBSeq. Statistically significant differential genes between K^+^ and Li^+^ conditions were determined using an FDR threshold of < 0.05.

### CLIP-seq data analysis

FASTQ files and BED files of C-terminal and full-length FUS-RNA binding sites from CLIP-seq in SH-SY5Y cells were downloaded from NCBI Accession GSE139263 [33]. BED files of FUS binding sites randomly distributed throughout the transcriptome were generated using bedtools shuffle.

### G4 predictions

Potential G4-forming sequences were identified transcriptome-wide with G4-iM Grinder [53] using cDNA and ncRNA FASTA files from Ensembl release 109 with the following parameters changed from default: DNA = FALSE, Complementary = FALSE, cGcC = TRUE, MinNRuns = 3.

### Biophysical assays

#### Oligoribonucleotides

Selected pG4 sequences (Table S1) were ordered from Eurogentec at 40 nmol scale, purified by reverse phase HPLC, from which a stock solution was prepared at 100 µM in RNase-free water.

#### CD

3 µM oligonucleotide was annealed in 10 mM lithium cacodylate pH 7.2 + 100 mM KCl or LiCl, by incubating at 65 °C for 3 min, then cooling to 25 °C. Spectra were measured from 220 to 335 nm at 25 °C, pathlength = 10 mm.

#### TDS

pG4 and mutant oligonucleotides were diluted in 10 mM lithium cacodylate buffer pH 7.2 + 100 mM KCl, and differential absorbance spectra between the unfolded and folded states were calculated after measuring the absorbance from 220 to 335 nm at 95 °C and 37 °C, respectively. Samples were held at 95 °C for 5 min prior to measurements to facilitate unfolding of secondary structures.

#### ThT and NMM fluorescence assays

Oligonucleotides for ThT and NMM fluorescence assays were prepared as described above for CD. Each sample was incubated with 2 μM of ThT or NMM for 10 min at 25 °C in 96-well plates with a final volume of 100 µL per well. Fluorescence was measured using a M1000 Pro (TECAN) monochromator plate reader with *λ*_ex_ = 420 nm, *λ*_em_ = 490 nm for ThT and *λ*_ex_ = 380 nm, *λ*_em_ = 610 nm for NMM. The measurements were performed with the following PMT settings: gain = 150 (fixed mode), integration time = automatic, excitation and emission slit width = 5 nm, number of flashes = 50, and Z-position = automatic.

#### EMSA

Selected pG4 sequences and their corresponding mutants were diluted to 5 µM in 25 µL of either 50 mM HEPES–KOH pH 7.4 + 200 mM KCl (“KCl” conditions) or 50 mM HEPES-LiOH pH 7.4 + 200 mM LiCl (“LiCl” conditions) supplemented with 5 mM EDTA. Structures were annealed by heating at 95 °C for 3 min followed by slow cooling to 25 °C. 1 µM of each annealed sequence was mixed with 0, 0.1, 1, or 5 µM of His_6_-FUS for 30 min at 30 °C in a Thermomixer (Eppendorf), with shaking (800 RPM), in 50 mM HEPES–KOH pH 7.4 + 150 mM KCl (“KCl” conditions) or 50 mM HEPES–LiOH pH 7.4 + 150 mM LiCl (“LiCl” conditions), supplemented with 200 ng BSA + 2 mM EDTA + 2 mM DTT and 5% glycerol. 6.25 pmol of His antibody (BioRad, 6200203) was then added and samples were incubated further at 30 °C for 30 min, with shaking (800 RPM). Ficoll (60 µg) was added and ~ 10.4 pmol of annealed sequence loaded onto a Novex 4–20% TBE precast gel (Invitrogen, EC6225BOX) pre-run in a cold room for up to 3 h in either TBE 0.5X + 50 mM KCl (“KCl” conditions) or TBE 0.5X + 50 mM LiCl (“LiCl” conditions) at 10 V/cm. Gels were run further for 3 h at constant voltage then stained for 30 min with SBYR Gold and imaged on a ChemiDoc MP imaging system (BioRad), using automated SYBR detection parameters.

## Supplementary Information

Additional file 1: Figs. S1–S9 and Tables S1 and S2.

## Data Availability

Raw sequencing reads of the G4 RIP-seq experiment performed in this article are deposited in the NCBI Gene Expression Omnibus (GEO) repository [GSE284272] [54]. Additional source data for this manuscript have been made available at Zenodo under a Creative Commons Attribution 4.0 International License (10.5281/zenodo.17069363) [55]. The FUS CLIP-seq dataset analysed for the current study, supporting the conclusions of this article is available in the NCBI GEO repository [GSE139263] [56].
