# Supplementary material for: G-quadruplex structural motifs modulate protein–RNA interactions within the transcriptome
Source: Genome Biol. 2025 Sep 30;26:324. doi: 10.1186/s13059-025-03795-0 (PMC12482648; doi:10.1186/s13059-025-03795-0)
Supplement: Supplementary file 1 — Additional file 1: Figs. S1–S9 and Tables S1 and S2. [file 13059_2025_3795_MOESM1_ESM.pdf]

Additional File 1

G-Quadruplex structural motifs modulate protein-RNA interactions within the transcriptome

Uditi Bhatt[1,2], Cameron W. Evans[1,2,3], Anne Cucchiarini[4], Julien Gros[4], K. Swaminathan Iyer[1,2], Jean-Louis Mergny[4,*] and Nicole M. Smith[1,2,*]

[1] School of Molecular Sciences, The University of Western Australia, Crawley, WA, 6009, Australia.

[2] ARC Training Centre for Next-Gen Technologies in Biomedical Analysis, The University of Western Australia, Crawley, WA 6009, Australia.

[3] School of Pharmacy and Pharmacology, University of Tasmania, Sandy Bay, TAS 7005, Australia

[4] Laboratoire d'Optique et Biosciences, École Polytechnique, CNRS, INSERM, Institut Polytechnique de Paris, 91120 Palaiseau, France.

* To whom correspondence should be addressed. Tel: +61 8 6488 4423; Email: nicole.smith@uwa.edu.au

Table of contents

Supplementary Figures.

Fig S1. 12% SDS-PAGE showing expression and purification of recombinant FUS protein in Rosetta2 (DE3) *E. coli.* S3

Fig S2. Pearson's correlation of RIP-seq biological replicates within K^+ (top) and Li^+ (bottom) RIP-seq samples. S4

Fig S3. Volcano plot of K^+ vs Li^+ RIP-seq Input samples. S5

Fig S4. K^+ vs Li^+ RIP-seq top genes log_2PostFC over gene length. S5

Fig S5. Heatmap of genes with $|log_2PostFC| > 0.585$. S6

Fig S6. Distribution of pG4s in RIP-seq data. S7

Fig S7. TDS spectra of wild type and mutant RNAs in K^+ conditions confirms formation of G4 structures. S8

Fig S8. CD spectra of RNAs in K^+ and Li^+ conditions. S9

Fig S9. ThT and NMM fluorescence for WT and mutant pG4 RNAs in K+ buffer conditions. S9

Supplementary Tables

Table S1. Sequences of pG4 RNA oligonucleotides to be used for *in vitro* biophysical assays and EMSA. S10

Table S2. Sequences of primers used for RT-qPCR. S11

Supplementary Figures

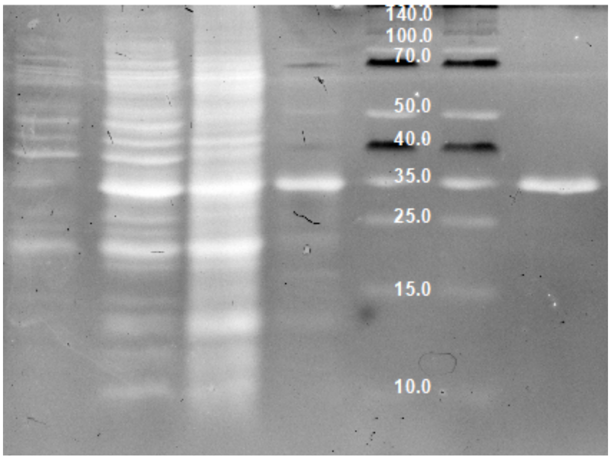

Fig S1. 12% SDS-PAGE showing expression and purification of recombinant FUS protein in Rosetta2 (DE3) *E. coli*. Lanes (left to right): 1 – total protein pre-IPTG induction, 2 – total protein post-IPTG induction, 3 – flow through, 4 – wash, 5 – ladder, 6 – ladder, 7 – 1 µg of purified protein, post-dialysis. Stained with Coomassie blue. Imaged on Amersham Typhoon.

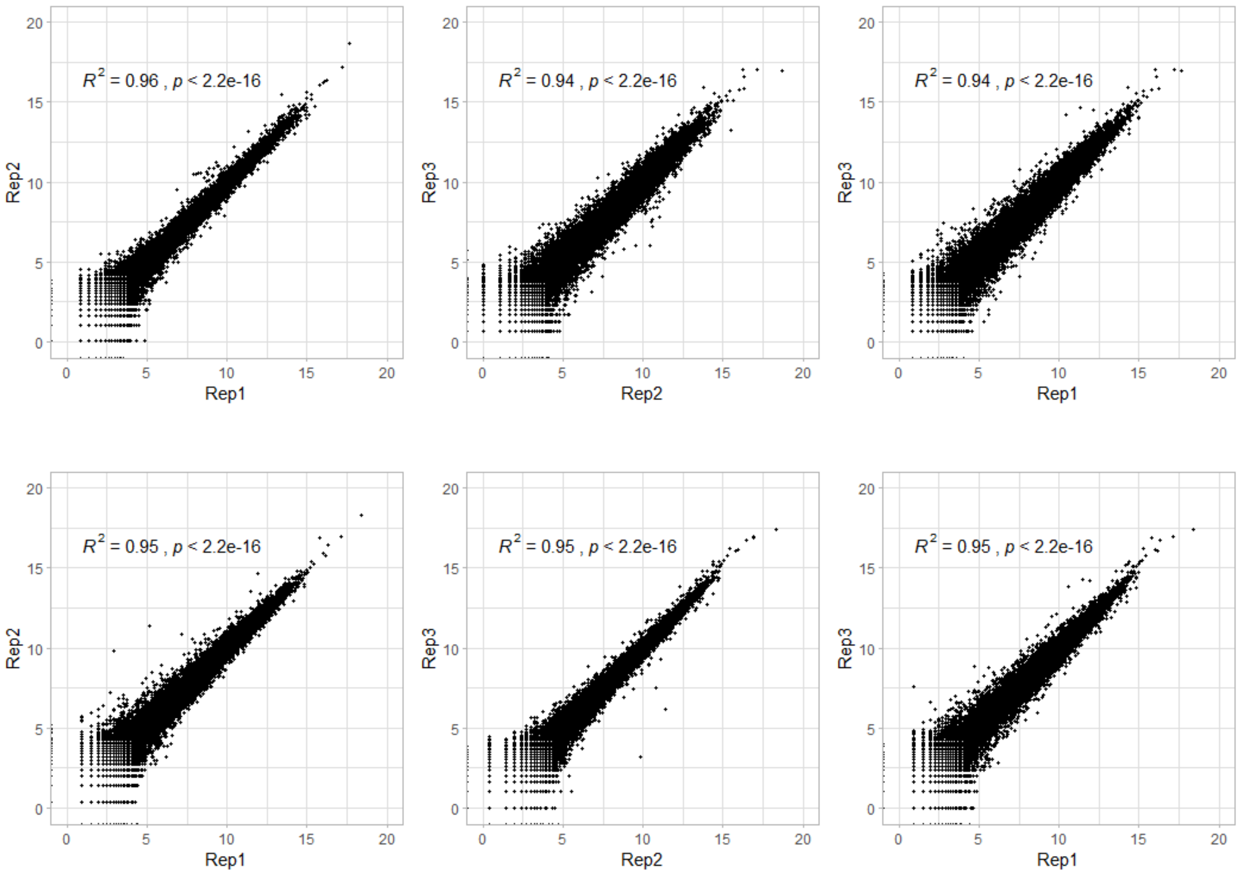

Fig S2. Pearson's correlation of RIP-seq biological replicates within K^+ (top) and Li^+ (bottom) RIP-seq samples. All axes represent read counts standardised to library size for each gene.

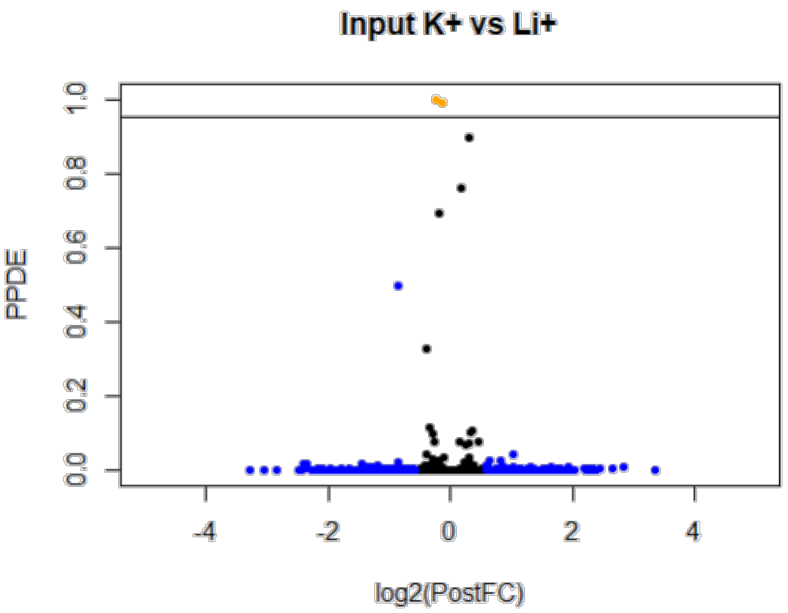

Fig S3. Volcano plot of K^+ vs Li^+ RIP-seq Input samples. Posterior probability of differential expression (PPDE) against $\log_2$PostFC. Horizontal line at 0.95 indicates statistically significant data points with FDR < 0.05 (orange). Genes with $|\log_2\text{PostFC}| > 0.585$ coloured blue.

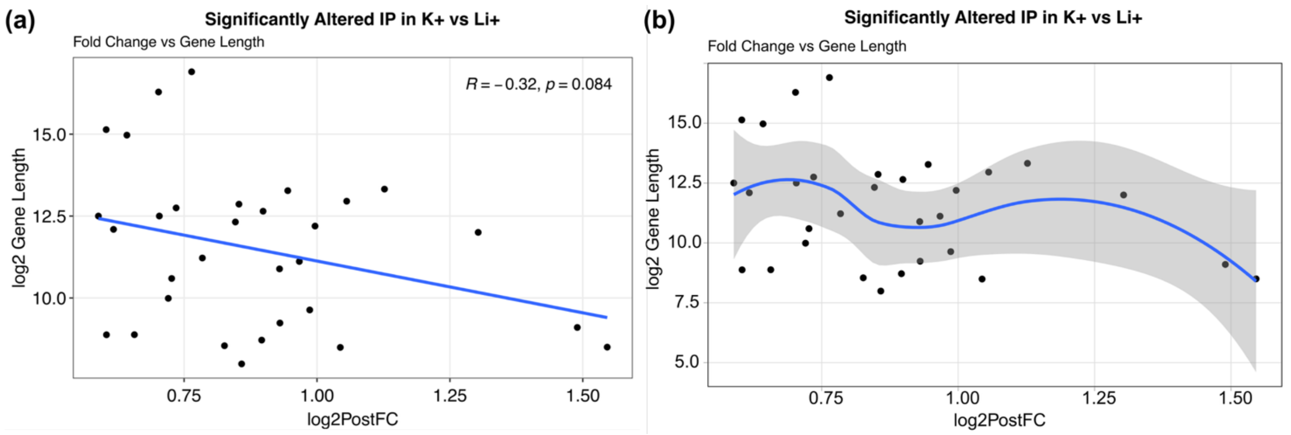

Fig S4. K^+ vs Li^+ RIP-seq top genes $\log_2$PostFC over gene length. No significant correlation between $\log_2$ Gene Length and $\log_2$PostFC observed for RNAs with significantly altered IP (FDR < 0.05, $|\log_2\text{PostFC}| > 0.585$) between K^+ and Li^+ conditions, shown as **(a)** line of best fit with Pearson's correlation ($R = -0.32$, $p = 0.084$) and **(b)** smoothed line with 95% confidence intervals.

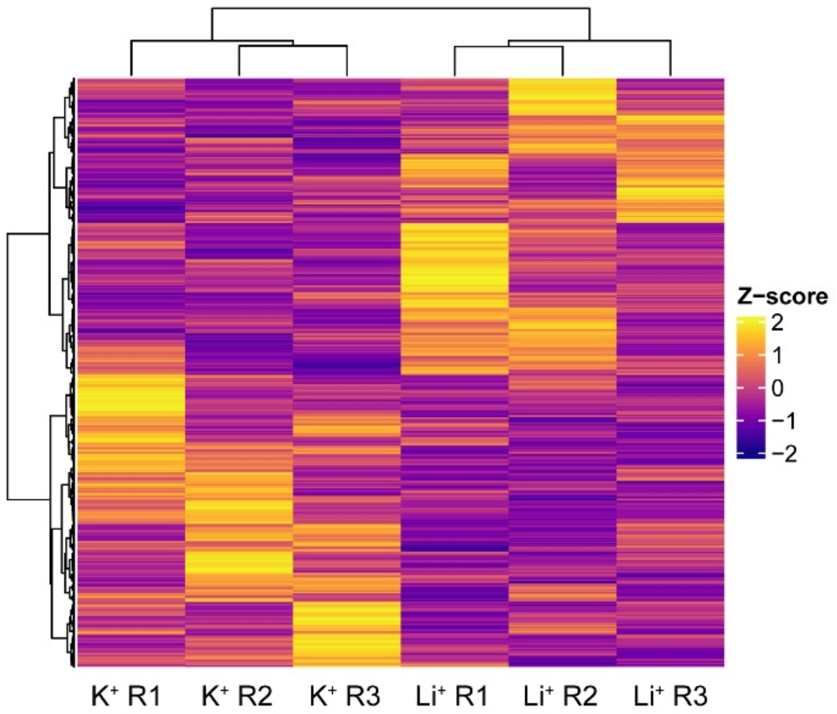

Fig S5. Heatmap of genes with $|\log_2 PostFC| > 0.585$.

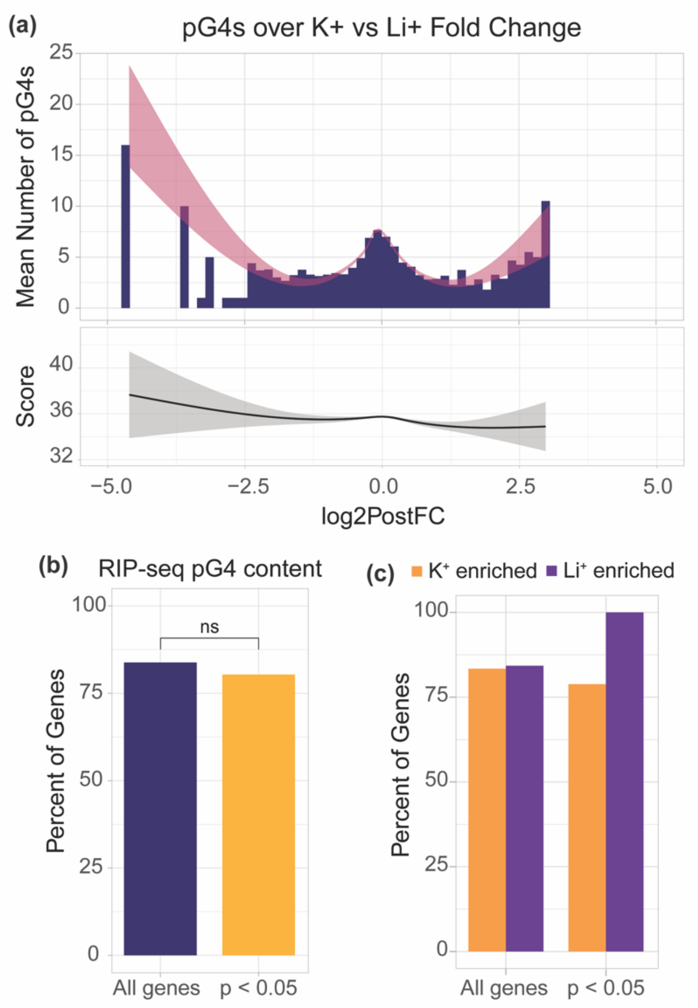

Fig S6. Distribution of pG4s in RIP-seq data. (a) Mean number (above) and score (below) of pG4s over $\log_2$PostFC between K^+ and Li^+ IP. Lines of best fit with 95% confidence intervals shown in pink and grey, respectively. Score = G4-iM Grinder score. **(b)** Proportion of genes containing at least one pG4 for the set of all genes (n = 23,520) and only genes with FDR < 0.05 (n = 56). **(c)** Proportion of genes containing at least one pG4 in K^+ and Li^+ conditions for all genes (n = 11,417 in K^+, 12,104 in Li^+) and significant genes (n = 53 in K^+, 3 in Li^+).

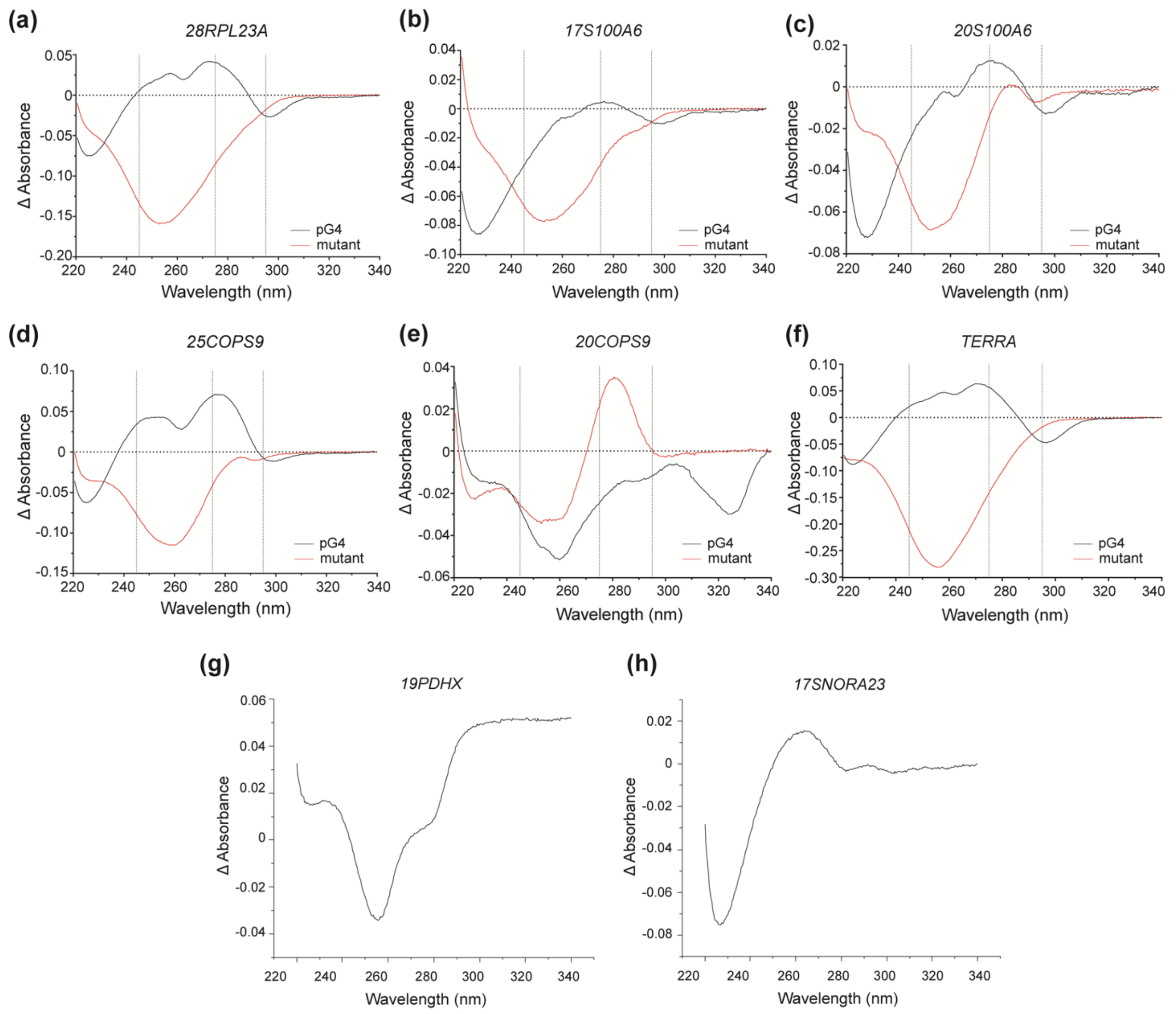

Fig S7. TDS spectra of wild type and mutant RNAs in K^+ conditions confirm formation of G4 structures. (a-e) G4-dependent FUS binding test RNAs, **(f)** positive control, and **(g,h)** negative controls.

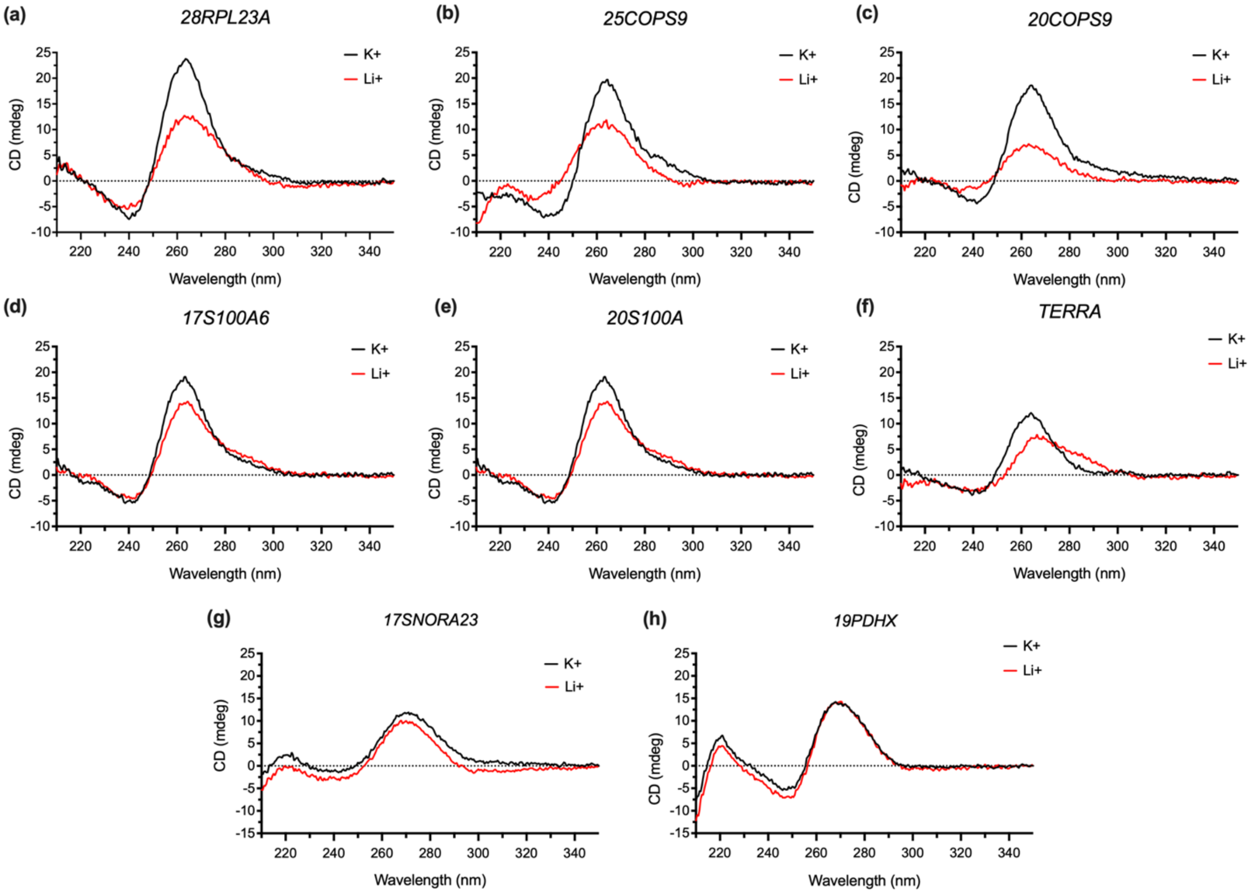

Fig S8. CD spectra of RNAs in K^+ and Li^+ conditions. Test pG4s **(a-e)** and positive control rG4 **(f)** show differential spectra between K^+ and Li^+, whereas negative control (g,h) spectra are largely similar between K^+ and Li^+.

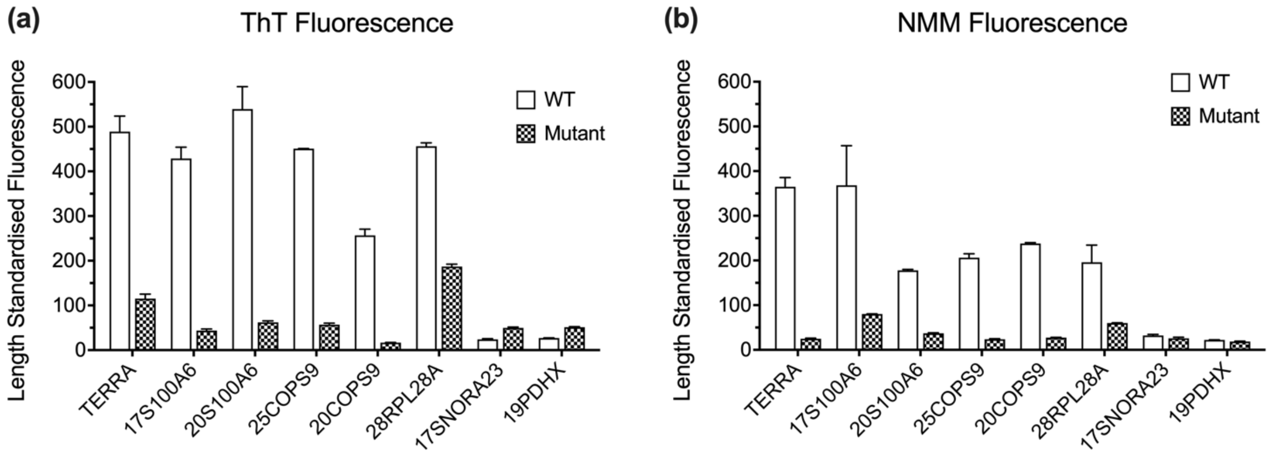

Fig S9. ThT and NMM fluorescence for WT and mutant pG4 RNAs in K^+ buffer conditions.

Supplementary Tables

Table S1. Sequences of pG4 RNA oligonucleotides to be used for *in vitro* biophysical assays and EMSA. Log_2PostFC indicates differential RNA immunoprecipitation between K^+ and Li^+ RIP-seq samples. For non-G4 forming mutant sequences, uracils that were converted from guanines are highlighted in bold and underlined. *TERRA* is used as a known FUS-binding rG4 positive control. *PDHX* and *SNORA23* are used as negative controls that do not contain a pG4 and were immunoprecipitated in both K^+ and Li^+ conditions or showed no pull-down, respectively. Distance indicates distance to the nearest FUS binding site identified from CLIP-seq data.

RNA	ENSEMBL Gene ID	Sequence	Log_2 PostFC	*p*-value	Distance (nt)
RPL23A	ENSG00000198242	AGGGGGAGGGUGUGGGGGCAGUGAGGGU	0.61	0	32
*RPL23A*_mut	–	AG**U**G**U**GAG**U**GUGUG**U**G**U**GCAGUGAG**U**GU	–	–	–
S100A6	ENSG00000197956	AGGGUGGGGCGCGGGCGGGA	0.58	6.47×10^{-13}	0
		UGGGCGGGCGGGGUGGG			0
*S100A6*_mut	–	AG**U**GUG**U**GGCGCG**U**GCG**U**GA	–	–	–
		UG**U**GCG**U**GCG**U**GGUG**U**G			–
COPS9	ENSG00000172428	UGGUGCGCGGGUCCCGGGGAGGGGA	1.12	0.001	0
		CGGGCGGGUGGGCGAUGGGC			0
*COPS9*_mut	–	UG**U**UGCGCG**U**GUCCCG**U**GGAG**U**GGA	–	–	–
		CG**U**GCG**U**GUG**U**GCGAUG**U**GC			–
TERRA	–	UUAGGGUUAGGGUUAGGGUUAGGG	–	–	–
PDHX	ENSG00000110435	ACUCUAACUAAUAAAGGAA	-0.003	1	–
SNORA23	ENSG00000201998	UCAUUCAGAUCUUGCUA	–	–	–

Table S2. Sequences of primers used for RT-qPCR. fwd = forward primer, rev = reverse primer.

Gene name	Sequence (5′ to 3′) / Catalogue #
NEAT1_2 fwd	GTCTTTCCATCCACTCACGTCTATTT
NEAT1_2 rev	GTACTCTGTGATGGGGTAGTCGTCAG
MALAT1 fwd	GGTCTCCCCACAAGCAACTT
MALAT1 rev	AACCCACCAAAGACCTCGAC
TERRA 17p fwd	CTTATCCACTTCTGTCCCAAGG
TERRA 17p rev	CCCAAAGTACACAAAGCAATCC
GAPDH	Qiagen #QT00079246
ACTB	Qiagen #QT01680476
